# The Growing Importance of the Human Biomonitoring of Exposure

**DOI:** 10.3390/ijerph17113934

**Published:** 2020-06-02

**Authors:** Giovanna Tranfo

**Affiliations:** Department of Occupational Medicine, Epidemiology, Occupational and Environmental Hygiene, INAIL Research, via Fontana Candida 1, Monte Porzio Catone, 00078 Rome, Italy; g.tranfo@inail.it

## 1. Introduction

The human biological monitoring of exposure is the determination of biomarkers, which can be dose biomarkers, measuring internal exposure levels to be compared with any (if known) biological limit value, effect biomarkers, which highlight early symptoms or dysfunctional situations still reversible with the improvement of the exposure situations, and susceptibility biomarkers, which express individual differences of genetic or acquired origin.

Biological monitoring can help to assess the health status in population groups or in occupational settings, complementary to the environmental monitoring, for the exposure assessment to chemical and biological risk agents.

This field has been slowly growing for many years but has recently had a strong acceleration thanks to the progress of analytical techniques. Besides, there is a greater sensitivity on environmental issues and on the impact on the health of the population. From the occupational point of view, the lowering of the exposure limits the use of personal protective devices and the attention to the skin absorbance have increased the importance of knowing the individual dose for each worker, and even the legislation has become aware of this need.

## 2. Population Studies

The best-known population study is the National Health and Nutrition Examination Survey (NHANES), a program of studies designed to assess the health and nutritional status of adults and children in the United States. The NHANES program began in the early 1960s and, in 1999, became a continuous program that has a changing focus on a variety of health and nutrition measurements to meet emerging needs. The survey examines a nationally representative sample of about 5000 persons each year. The program involves the collection of blood and urine samples and the analysis of about 60 parameters, including heavy metals, volatile organic compounds, pesticides, phthalates, hormones, viruses [1].

HBM4EU is a five-year project coordinating and advancing human biomonitoring in Europe that was kicked off in 2017 and will run to the end of 2021; it is a joint effort of 30 countries, the European Environment Agency and the European Commission, co-funded under Horizon 2020. HBM4EU is generating evidence of the actual exposure of citizens to chemicals and the possible health effects in order to support policymaking. Partners effectively communicate results to policy makers, ensuring their exploitation in the design of new chemicals policies and the evaluation of existing measures. This initiative contributes directly to the improvement of health and well-being for all citizens, by investigating how exposure to chemicals affects the health of different vulnerable groups, such as children and pregnant women, as well as of highly exposed groups, such as workers [2].

It is necessary to promote biological monitoring studies in the general population to reveal which levels of substances are increasing or decreasing over time and to provide guidance for policy actions. These levels, called biological guidance values (BGVs), are the concentration of a certain substance or its metabolite in an appropriate biological medium corresponding to a certain percentile (e.g., 95th percentile) in a defined reference population. If it is not possible to detect the background levels, the BGV can be equivalent to the detection limit of the analysis method, which must therefore be specified. For those pollutants that are present both in the environment and in the workplaces, these values could also provide the reference value (RV) for the interpretation of the data measured in the workers.

## 3. Occupational Studies and European Regulations

The use of human biomonitoring in the assessment of occupational exposures involves the comparison with biological limit values. Health-based biological limit values (BLVs) are reference values for evaluating potential health risks in the practice of occupational health. Due to biological variability, an individual’s measurement may exceed the BLV without incurring an increased health risk. If, however, the biological levels persistently exceed the BLV, the cause of the excessive values must be investigated and proper action taken to reduce the exposure. BLVs represent the levels of determinants that are most likely to be observed in specimens collected from a worker exposed to the chemical in question, exclusively by inhalation, at the level of the occupational exposure limit (OEL). Exceptions are BLVs for substances for which the OELs serve as protection against non-systemic effects (e.g., irritation or respiratory disorders) or for substances which require biological monitoring due to other routes of absorption, in particular the skin. For such substances, the monitoring of concentrations in ambient air may not be sufficient, and biological monitoring strategies appear to be of potential importance in the medical surveillance of exposed workers.

The American Conference of Governmental Industrial Hygienists (ACGIH) published and updates yearly a list of more than 50 BEIs (biological exposure indices) that cover more than 80 chemical substances, which are guidance values for assessing biomonitoring results, and represent levels of determinants most likely to be observed in samples collected from healthy workers with inhalation exposure at the OEL [3].

The International Committee of Occupational Health (ICOH), an international non-governmental professional society whose aims are to foster the scientific progress, knowledge and development of occupational health and safety in all its aspects, is recognized by the United Nations as a non-governmental organization (NGO) and has close working relationships with International Labour Office (ILO) and WHO. The ICOH Scientific Committee of Occupational Toxicology (SCOT) founded in 1990 has the mission of promoting research in all areas of occupational toxicology. A current focus of SCOT is the use of biological monitoring in exposure assessment and intervention in workplaces and the general environment, based on solid scientific and ethical principles. This has achieved bringing together the world’s leading experts and practitioners to discuss and share knowledge on new biomarkers, new analytical techniques, case studies of occupational and environmental exposures and the development of policies and guidance to use, interpret and manage biological monitoring as a tool to identify, monitor and control chemical exposures [4].

The European Council Directive 98/24/EC of 7 April 1998 gave a definition of Biological limit value as “the limit of the concentration in the appropriate biological medium of the relevant agent, its metabolite, or an indicator of effect”; the first binding biological limit value to be established was that for Lead and its inorganic compounds. The SCOEL (Scientific Committee for Occupational Exposure Limits) of the European Union had proposed biological limit values for 22 substances [5], but, since then, no more binding biological limit values were established, even if many EU countries have set national limits. However, biological monitoring has been indicated as appropriate when the skin absorption feature of the substances is noted, in the Chemical Agents Directives establishing indicative occupational limit values. In 2018, the RAC (Risk Assessment Committee of European Chemical Agency (ECHA)) recommended a new, lower OEL for benzene, together with both two BLVs and two BGVs: for an 8 hour TWA OEL of 0.05 ppm, a BLV of 0.7 µg benzene/L urine or 2 µg S-phenylmercapturic acid (SPMA)/g creatinine and a BGV of 0.3 µg benzene/L urine or 0.5 µg S-phenylmercapturic acid (SPMA)/g creatinine [6].

Recently, the EU Directive 2019/983 also stated that the Commission should issue practical guidelines for biological monitoring.

## 4. New Biomarkers Discovery by Metabolomics

The research of new biomarkers is a continuous task in many disciplines, including human and animal health, biomarker discovery, drug discovery and development, plant biology, microbiology, food chemistry and environmental monitoring. The identification and quantification of all metabolites in human biological fluids has been made possible by untargeted metabolomics, an emerging ‘omics’ science involving the characterization of metabolites in biological systems [7].

The ability of metabolomics analysis to characterize and capture the overall influence of net chemical mixtures (including interactive effects) and spatial/temporal heterogeneity in contaminant distribution on living organisms draws attention to its future value and use in the emerging field of environmental risk assessment [8].

In the occupational field, the discovery of new biomarkers, especially effect biomarkers, will permit us to measure the health effect of occupational exposures, even in conditions that are regarded as not dangerous, when workers’ exposure values are below the occupational exposure limits, like in the case of chronic exposure to very low levels of xenobiotics, or in the case of exposure to mixtures of several substances.
